# Curcumin from Turmeric Rhizome: A Potential Modulator of DNA Methylation Machinery in Breast Cancer Inhibition

**DOI:** 10.3390/nu13020332

**Published:** 2021-01-23

**Authors:** Krystyna Fabianowska-Majewska, Agnieszka Kaufman-Szymczyk, Aldona Szymanska-Kolba, Jagoda Jakubik, Grzegorz Majewski, Katarzyna Lubecka

**Affiliations:** 1 Faculty of Medicine, Lazarski University, 02-662 Warsaw, Poland; krystyna.fabianowska-majewska@lazarski.pl (K.F.-M.); grzegorz.majewski@lazarski.pl (G.M.); 2 Department of Biomedical Chemistry, Faculty of Health Sciences, Medical University of Lodz, 92-215 Lodz, Poland; agnieszka.kaufman-szymczyk@umed.lodz.pl (A.K.-S.); szymanska.aldona@gmail.com.pl (A.S.-K.); jagoda.jakubik@stud.umed.lodz.pl (J.J.)

**Keywords:** curcumin, turmeric, bioactive nutraceutical, nutriepigenomics, DNA methylation, breast cancer, chemoprevention

## Abstract

One of the most systematically studied bioactive nutraceuticals for its benefits in the management of various diseases is the turmeric-derived compounds: curcumin. Turmeric obtained from the rhizome of a perennial herb *Curcuma longa* L. is a condiment commonly used in our diet. Curcumin is well known for its potential role in inhibiting cancer by targeting epigenetic machinery, with DNA methylation at the forefront. The dynamic DNA methylation processes serve as an adaptive mechanism to a wide variety of environmental factors, including diet. Every healthy tissue has a precise DNA methylation pattern that changes during cancer development, forming a cancer-specific design. Hypermethylation of tumor suppressor genes, global DNA demethylation, and promoter hypomethylation of oncogenes and prometastatic genes are hallmarks of nearly all types of cancer, including breast cancer. Curcumin has been shown to modulate epigenetic events that are dysregulated in cancer cells and possess the potential to prevent cancer or enhance the effects of conventional anti-cancer therapy. Although mechanisms underlying curcumin-mediated changes in the epigenome remain to be fully elucidated, the mode of action targeting both hypermethylated and hypomethylated genes in cancer is promising for cancer chemoprevention. This review provides a comprehensive discussion of potential epigenetic mechanisms of curcumin in reversing altered patterns of DNA methylation in breast cancer that is the most commonly diagnosed cancer and the leading cause of cancer death among females worldwide. Insight into the other bioactive components of turmeric rhizome as potential epigenetic modifiers has been indicated as well.

## 1. Introduction

The rates of incidence and mortality from female breast cancer are swiftly increasing worldwide. The variety of causes of mammary cancer reflects the heterogeneity, extended life expectancy, and rapid expansion of the global population. Thus, the differences in the prevalence of the risk factors for breast cancer are often associated with socioeconomic development, followed by deleterious lifestyle changes and environmental exposures, including diet, that are major determinants of any type of cancer. Mammary cancer is the most commonly diagnosed cancer and the leading cause of cancer death among females [1,2]. According to Global Cancer Statistics (GLOBOCAN) 2018, the estimates of incidence and mortality worldwide for breast cancer show about 2.1 million newly diagnosed female breast cancer cases, accounting for almost 25% of all cancer cases, as well as over 626,000 deaths, accounting for 15% of all cancer deaths among women [1,2].

Among various anti-cancer strategies, the early detection (screening and surveillance) remains the best approach to enhance and manage the mammary cancer outcomes, although adherence to the breast cancer screening guidelines is still low. Thus, the application of different therapeutics is still an effective therapy against breast cancer. As over 70% of breast cancer cases are estrogen receptor (ER) positive type, hormonal therapy or aromatase inhibitors are often used as the main treatment. However, there is another group of triple-negative breast cancer (TNBC) patients that lack the expression of ER, PR (PRGR; Progesterone receptor), and HER-2 (ERBB2; Receptor tyrosine-protein kinase erbB-2) receptors. Five subtypes of breast cancer (Luminal A, Luminal B, basal, EERB2-overexpressing, and normal breast-like subtypes) have been described based on the gene expression profile [3,4]. Since breast cancer comprises a heterogeneous population of cells (breast cancer heterogeneity) [3,4], it makes it more difficult to treat with the current standard of therapy, including surgery, radiation, and chemotherapeutic drugs [5]. In cancer cells, including mammary cancer cells, diverse genetic and epigenetic alterations, simultaneous activation of numerous cell-surface receptors and interconnected, multiple, complex signaling pathways have been observed [6]. Hence, using anti-cancer drugs targeting a single gene product or cell signaling pathway from the whole intricate cancer-related signaling network may lead to activation of alternative pathways followed by the development of drug resistance and tumor recurrence that are common in all breast cancer subtypes [3,4,5,6]. All this, together with the high toxicity of the current conventional single-target chemotherapeutics [5] entails the necessity of finding novel anti-cancer agents with low toxicity and enhanced efficacy, targeting multiple cancer-related genes and signaling pathways. 

The personal or family history of mammary malignancies and inherited genetic mutations in breast cancer susceptibility genes, mostly BRCA1 (BRCA1 DNA Repair Associated) and BRCA2 (BRCA2 DNA Repair Associated), account for 5% to 10% of breast tumor cases. The studies of people migration have revealed that nonhereditary factors (i.e., demographic, social, economic, environmental, and lifestyle factors) are the main drivers of the observed international and interethnic differences in cancer incidence, including breast cancer incidence. Increased incidence rates of breast cancer in successive generations of the developed countries with higher HDI (The Human Development Index) and transitioned countries are attributed to a raised prevalence of known risk factors [1,2]. Those risk factors are related to menstruation (early menarche, late menopause), reproduction (nulliparity, the postponement of childbearing, having fewer children), exogenous hormone intake (prolonged oral contraception and hormone replacement therapy), nutrition (poor diet with excessive consumption of processed meat and red meat, alcohol abuse), anthropometry (overweight in adulthood, greater levels of obesity, mass and distribution of body fat), cigarette smoking, and physical inactivity. Thus, breastfeeding (with longer duration), healthy diet, and physical activity are known preventive factors with potential beneficial health effects [7].

Therefore, it is necessary to ask why certain types of cancer, such as breast cancer, are more prevalent in some countries than in others. The incidence rate of breast neoplastic diseases is predominantly higher in women of Western nations when compared to women in Asian countries. The exact reason for this disparity is not clear, but dietary factors have been conceived to account for approximately 30% of cancer cases in Western nations [8,9]. It has been hypothesized that different dietary patterns related to specific culture and ethnicity, including consumption of a variety of vegetables, herbs and spices abundant in natural bioactive compounds (i.e., polyphenols and vitamins) would be a pivotal reason [8,9]. Of all the spices, turmeric (*Curcuma longa* L.) has been gaining more and more attention due to its beneficial health effects. The turmeric-derived polyphenol, curcumin, is one of the most systematically studied bioactive nutraceuticals for its utility in the management of various diseases, with cancer at the forefront [10]. Importantly, recent reports have shown that some phytochemicals, such as curcumin have the ability to target many breast cancer-related signaling pathways, including Wnt/β-Catenin, Notch, Hedgehog, JAK-STAT, and PI3K/Akt/mTOR [11]. The hitherto studies on curcumin chemopreventive activities suggests that curcumin is one of the most relevant compounds to manage the challenges of breast cancer treatment [10,11,12].

Moreover, curcumin is well known for its potential role in inhibiting cancer by targeting epigenetic machinery, especially DNA methylation machinery. The dynamic DNA methylation processes serve as an adaptive mechanism to a wide variety of environmental factors, including diet. Every healthy tissue has a precise DNA methylation pattern that changes during cancer development forming a cancer-specific design. Hypermethylation of tumor suppressor genes (TSGs), global DNA demethylation, and hypomethylation of oncogenes and prometastatic genes are hallmarks of nearly all types of cancer, including breast cancer. Curcumin has been shown to modulate epigenetic events that are dysregulated in cancer cells and possess the potential to prevent cancer or enhance the effects of conventional anti-cancer therapy [12,13].

Although mechanisms underlying curcumin-mediated changes in the epigenome remain to be fully elucidated, the mode of action targeting both hypermethylated and hypomethylated genes in cancer is promising for cancer chemoprevention.

This review provides a comprehensive discussion of potential epigenetic mechanisms of curcumin in reversing altered patterns of DNA methylation in breast cancer. Insight into the other bioactive components of turmeric rhizome as potential epigenetic modifiers has been indicated as well. The turmeric rhizome contains not only curcumin but also other antioxidative agents, such as C and E vitamins, several minerals, as well as B-group vitamins (i.e., B2, B6 and B9 vitamins) participating in one-carbon metabolism via regulation of S-adenosyl-L-methionine (SAM, a ubiquitous methyl group donor) pool and DNA methylation reaction.

## 2. Curcumin: Chemical Structure and Physical Properties

Curcumin (synonym: diferuloylmethane; molecular formula: C_21_H_20_O_6_; molecular weight: 368.38 g/mol) is a well-known dietary polyphenol (IUPAC name: 1,7-bis(4-hydroxy3-methoxyphenyl) hepta-1,6-diene-3,5-dione) derived from the rhizome of turmeric, *Curcuma longa* L. [14] and other *Curcuma species* of the ginger family, Zingiberaceae. Both turmeric and curcumin have a history of human application in foods, supplements, and cosmetics, as well as for therapeutic goals in Asia, Europe and the United States of America. Products containing curcumin are available on the market all over the world. The bright orange-yellow powder known as turmeric is prepared from boiled and dried rhizomes of the Asian, perennial, herbaceous plant *Curcuma longa* L. It includes a mixture of three diarylheptanoids, together called curcuminoids, i.e., curcumin, demethoxycurcumin, and bis-demethoxycurcumin [12,15]. The literature data indicates the level of curcumin and other curcuminoids in turmeric powder at approximately 3–5% [16]. Commercially, in turmeric extract, mostly used in preclinical studies and clinical trials, the curcuminoid content is often increased to as high as 95%, including approximately 75% (a/a) curcumin, 20% (a/a) demethoxycurcumin, and 5% (a/a) bisdemethoxycurcumin (HPLC, area%). These phytochemicals are practically insoluble in water at acidic and neutral pH, but soluble in methanol, ethanol, acetone and dimethylsulfoxide (DMSO).

The curcumin itself is a colored, yellow to orange, crystalline compound that is commonly used as a coloring and flavoring agent, and food additive. In 2004, the Joint FAO (The Food and Agriculture Organization)/WHO (The World Health Organization) Expert Committee on Food Additives (JECFA) established an acceptable daily intake (ADI) for curcumin of 0–3 mg/kg body weight. The ADI estimation for curcumin was based on the NOAEL (no-observed-adverse-effect level) of 250–320 mg/kg body weight/day from the reproductive toxicity study for a decreased body weight gain in the F2 rat generation observed at the maximum dose, and an uncertainty factor equal to 100 [17]. JECFA and the European Food Safety Authority (EFSA) Panel on Food Additives and Nutrient Sources added to Food (ANS)) agreed that curcumin (symbol E-100, EFSA) is not carcinogenic and genotoxic. The EFSA Panel perceived that the normal diet provides the curcumin amount of less than 7% of the aforementioned ADI [18].

Curcumin is a hydrophobic molecule, practically insoluble in the aqueous phase of the digestive fluids. Curcumin is rapidly eliminated from the digestive tract, having poor oral bioavailability, due to low absorption from the intestine and rapid degradation in the liver [19] reported in human and animal studies [15]. Under physiological pH conditions, such as 0.1 M phosphate buffer (pH 7.2) at 37 °C over 90% of curcumin is degraded within 30 min [20]. The in vivo studies indicate that following curcumin reduction to dihydrocurcumin and tetrahydrocurcumin, quick conversion to mono-glucuronidated conjugates occurs [21]. Thus, the main curcumin metabolites reported in vivo are the curcumin-, dihydrocurcumin-, and tetrahydrocurcumin-glucuronides, as well as tetrahydrocurcumin [22].

Moreover, the studies in humans revealed that it is unlikely that substantial concentrations of curcumin occur in the body after its ingestion at high doses up to 12 g/person, equivalent to 200 mg/kg body weight for a 60 kg individual. Even upon the oral exposure of 10–12 g of curcumin, the detected plasma concentration of this polyphenol was low, in the nanomolar range, with the highest level of less than 160 nmol/L [23]. The numerous studies supported the safety of high doses of curcumin, depicting only gastric disturbance [15]. 

According to reports of the U.S. Department of Agriculture (USDA), 100 g of turmeric rhizome contain from 2% to 9% of curcumin and other curcuminoids, as well as several vitamins. Those vitamins include some lipid-soluble vitamins such as E and K vitamins, as well as some water-soluble vitamins such as vitamin C and the B-group vitamins, i.e., folic acid (B9), riboflavin (B2), and pyridoxine (B6). Moreover, the *Curcuma longa* rhizome comprises the minerals (Fe, Mg, Zn, K, Na, and Ca) and macromolecules (proteins, lipids, carbohydrates, and dietary fiber) essential for human health (Table 1) [19]. The co-occurrence of several bioactive compounds such as curcumin and vitamins C and E in natural turmeric enhances its antioxidative properties and encourage greater consumption of natural Curcuma-derived products rather than dietary supplements with pure turmeric-extracted curcumin.

Due to the poor bioavailability and low absorption of pure bioactive curcumin, many researchers have focused on studies to ameliorate its bioavailability, pharmacological properties, chemopreventive activity and therapeutic utility [24,25,26]. The novel curcumin derivatives have been developed. Thus, curcumin and its derivatives have been still extensively investigated as potential anti-cancer, antioxidant, anti-bacterial, anti-inflammatory, analgesic, accelerating wound healing and improving digestion processes agents. The recent studies have revealed that bioavailability of pure curcumin may be enhanced by various natural or synthetic adjuvants, i.e., piperine from black pepper [24] or folic acid [25]. Moreover, taking into account curcumin hydrophobic properties, its bioavailability and retention time can be improved by applying different forms of conjugates, including liposomes, polymeric micelles, phospholipid complexes, microemulsions and nanoparticles [26]. Further studies are needed to establish their clinical application and effectiveness.

## 3. DNA Methylation and Demethylation Processes 

DNA methylation is one of the most important, epigenetic modifications without any alterations in the primary DNA sequence and is frequently associated with silencing of gene expression [27]. This process is tightly connected with replication during the normal cell growth and plays an important role in the regulation of crucial cell functions such as DNA repair, cell cycle, cell differentiation, intracellular signal transduction, and cell apoptosis. Moreover, the aberrations of DNA methylation patterns can be implicated in neoplastic processes of both normal cells and cells with pathological changes. The aberrant DNA methylation patterns are observed at very early stages of pre-cancerous transformation of cells that are still not exhibiting any cancerous phenotype [28]. The tumor-specific alterations of DNA methylation pattern can include global DNA hypomethylation and loci-specific hypomethylation of oncogenes and pro-metastatic genes, as well as loci-specific hypermethylation within TSG regulatory regions such as proximal promoter regions frequently containing CpG islands and/or enhancers [28,29,30,31]. 

The ageing or cancer-related global DNA hypomethylation may result in microsatellite instability, transposon activation and stimulation of oncogene expression. In cancer, the hypermethylation of TSG promoters is often associated with the overexpression of DNA methyltransferases (DNMTs). The DNMT enzymes catalyze a reaction in which methyl group is transferred from S-adenosyl-L-methionine (SAM) to cytosine located in CpG dinucleotide sequences (CpGs) giving 5-methylcytosine (5mC). In normal cells, CpG-rich regions, called CpG islands, are mostly unmethylated and are located within regulatory regions of house-keeping genes, tissue-specific genes, and TSGs [32]. The elevated promoter methylation is a predominant mechanism of chromatin inactivation. Epigenetic modifications, apart from DNA methylation, include covalent post-translational modifications of histone tails (mainly methylation/demethylation and acetylation/deacetylation). DNA hypermethylation and histone deacetylation are concerted to determine the transcriptional activity of certain genes, leading frequently to condensation of chromatin structure making the DNA inaccessible to complexes of transcription proteins [33].

Neoplastic development might be associated with both hypermethylation of gene promoters and increase in the number of silent genes, mainly TSGs encoding proteins that control the normal cell functions and the balance between cell proliferation and apoptosis. Additionally, DNA hypermethylation of CpG island within the gene promoter can cause genetic alterations. The spontaneous deamination of 5mC and its transition to thymine within CpG sequences of *TP53* (*Tumor Protein P53*) gene have been observed in cells of various types of cancer [34].

In mammalian cells, the reaction of DNA methylation is the post-replicative DNA modification taking place at the replication fork. It is catalyzed by the enzymes of the DNMT family, including DNMT1, DNMT2, DNMT3A, DNMT3B, and DNMT3L (DNA Methyltransferase 3 Like) [32]. DNMT1 is the main DNA methylating enzyme responsible for the maintenance DNA methylation in normal cells, as well as for the maintenance and de novo DNA methylation in cancer cells. DNMT3A and DNMT3B catalyze de novo DNA methylation. The differences between the DNMT1 and other DNMTs are in the length of the N-terminal regulatory domain. The C-terminal catalytic domain of DNMT1 protein contains the following regions: one implicated in the binding of SAM (methyl donor), another one responsible for binding to DNA, and an active center containing proline and cysteine. Whereas, the N-terminal regulatory domain of DNMT1 can interact with numerous proteins like DMAP1 (DNA methyltransferase 1-associated protein 1), PCNA (Proliferating cell nuclear antigen), and RB (Retinoblastoma-associated protein). It is multifunctional and contains a DNA binding region, a cysteine-rich region, several Zn-binding domains, and two regions responsible for the localization to replication foci. There are also regions implicated in DNMT1 interactions with histone deacetylases HDAC1 and HDAC2, as well as the other DNA methyltransferases, DNMT3A and DNMT3B [13].

In mammalian cells, two additional DNMTs have been described, i.e., DNMT2 (TRDMT1) and DNMT3L. They do not possess catalytic activity towards DNA. The DNMT3L has been shown to interact with de novo DNMTs, DNMT3A and likely DNMT3B, what supports their stability and stimulates DNMT3A-mediated DNA methylation [35]. The DNMT2 activity does not involve DNA methylation but RNA methylation, specifically cytosine 38 in the anticodon loop of aspartic acid tRNA [36]. The role of DNMT3L in breast tumorigenesis is not well understood. Girault et al. reported that DNMT3L mRNA levels were very low (only detectable but not quantifiable) in a subgroup of 46 breast tumors [37].

Upon DNA replication, within the transcriptionally active DNA (euchromatic DNA), the DNMT1 cooperates with the PCNA protein to form the DNA-DNMT1-PCNA complex responsible for the maintenance of the DNA methylation patterns [38]. In normal somatic cells, the DNA methylation can be inhibited by CDN1A (Cyclin-dependent kinase inhibitor 1, encoded by *CDKN1A* (*P21*) gene) protein, which is the inhibitor of cyclin-dependent kinases. In this case, the CDN1A (P21) protein disrupts the PCNA-DNMT1 complex and forms a new P21-PCNA complex. It leads to inhibition of polymerase δ activity followed by inhibition of both DNA methylation and replication [39]. All this allows the repair processes of DNA double-strand breaks. It is noteworthy to indicate that the P21 and DNMT1 proteins compete for binding to the same motif of the PCNA protein. Several studies have revealed an inverse relation between P21 and DNMT1 concentrations in normal and cancer cells. In normal cells, the protein level of P21 is much higher than DNMT1, whereas in cancer cells the relation is opposite [40].

In normal cells, the DNMT1 activity can be also inhibited by interaction of RB protein with the N-terminal regulatory domain of the DNMT1 protein. The RB binding to DNMT1 blocks the formation of the DNA-DNMT1-PCNA complex and consequently DNA methylation reaction. It might be one of the mechanisms preventing some DNA fragments (e.g., promoters of TSGs) from methylation during normal cell development [41]. 

The DMAP1 (transcriptional co-repressor) protein affects DNMT1 activity as well. It interacts with DNMT1 at replication foci when the DNMT1 enzyme is bound to PCNA. During this interaction, DNMT1 binds to the co-repressor DMAP1 and simultaneously to HDAC2. The repressive activity of this complex is probably responsible for the appearance of the condensed chromatin state (heterochromatin) and the maintenance of transcriptional silencing of methylated genes upon DNA replication [42].

Among important proteins interacting with the regulatory domain of DNMT1 are the nuclear proteins that are characterized by the presence of a methyl-CpG binding domain (MBD). The MBD2 (Methyl-CpG-binding domain protein 2), MBD3 (Methyl-CpG-binding domain protein 3) and MECP2 (Methyl-CpG-binding protein 2) proteins exert a two-way effect on the epigenome. On the one hand, MECP2 can bind fully methylated DNA mediating transcriptional repression through interaction with HDAC and the corepressor SIN3A (Paired amphipathic helix protein Sin3a) [43]. On the other hand, MBD proteins can bind to hemimethylated DNA and then form the complex with DNMT1 that is forced to catalyze methylation of a newly synthesized DNA strand and cover recognition elements [44].

To achieve transcriptional silencing of the selected genes, DNMT1 cooperates directly with de novo DNMTs, DNMT3A and DNMT3B. These two enzymes are responsible for the de novo DNA methylation in normal cell development. However, in cancer cells, DNMT3A and DNMT3B enzymes synergistically enhance the DNMT1 activity and shift its activity towards de novo DNA methylation [40].

Moreover, the studies revealed that DNMT1 directly interacts with histone-modifying enzymes such as histone deacetylases HDAC1 and HDAC2, and histone methyltransferase SUV91 (Histone-lysine N-methyltransferase SUV39H1) [45]. The studies revealed that methylation of heterochromatic DNA might be triggered by histone modifications, i.e., methylation of histone H3, and deacetylation of histone H3 and H4 [46].

Thus, the interactions of DNMT1 with the aforementioned proteins, i.e., MBPs, DNMT3A and DNMT3B, HDAC1 and HDAC2, and SUV39B may result in transcriptional repression of many genes in cancer cells, mainly TSGs, thereby stabilizing the heterochromatic state [32]. The aberrant DNMTs activity followed by the altered DNA methylation patterns plays a pivotal role during carcinogenesis, including breast cancer development. The literature data indicated that more than 100 genes have been hypermethylated in primary breast tumors and breast cancer cell lines, i.e., *PTEN* (Phosphatase And Tensin Homolog), *RARB* (Retinoic Acid Receptor Beta), *APC* (APC Regulator Of WNT Signaling Pathway), *CDKN2A* (*P16*; Cyclin Dependent Kinase Inhibitor 2A), *CDH1* (Cadherin 1), *DAPK1* (Death Associated Protein Kinase 1), *GSTP1* (Glutathione S-Transferase Pi 1), *RASSF1* (Ras Association Domain Family Member 1), *TIMP3* (TIMP Metallopeptidase Inhibitor 3), and *MGMT* (O-6-Methylguanine-DNA Methyltransferase) [13,47].

The role of altered expression of TET (Methylcytosine dioxygenase TET) demethylating enzymes (i.e., TET1, TET2 and TET3) in cancer is less well understood. The downregulation of TET expression and reduced 5hmC (5-hydroxymethylcytosine) levels have been shown to be associated with breast, gastric, liver, and lung tumors [48]. Moreover, high levels of TET expression estimated in over 160 samples of breast cancer tissue correlated with increased patient survival, probably resulting from potential DNA demethylation–mediated upregulation of TSGs [49]. Yang et al. reported that the TETs expression, mostly TET1, was significantly decreased in human breast tumors, and the 5hmC levels were broadly diminished in breast cancer tissues [50].

**Figure 1:**
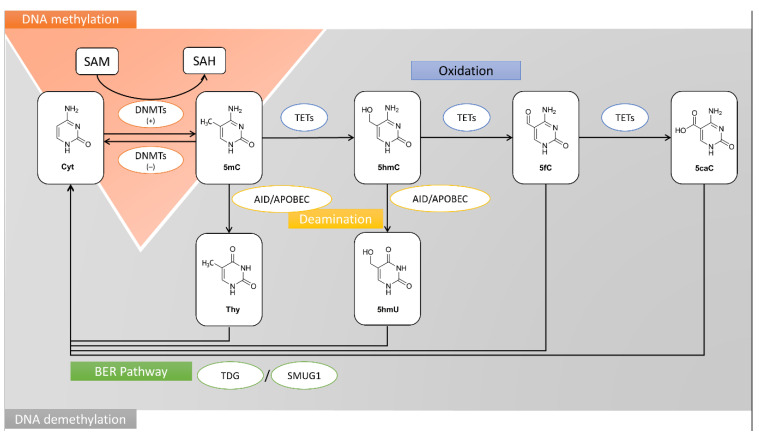
The mechanisms of DNA methylation and demethylation processes. SAM is a methyl donor in the DNMT-catalyzed methylation reactions of cytosine (Cyt) to 5mC. TET proteins catalyze the multi-stage process of 5-methylcytosine oxidation to 5hmC, 5fC, and 5caC. Furthermore, 5mC and 5hmC may be transformed into thymine (Thy) and 5hmU in deamination process, respectively. These misincorporations are recognized and replaced with cytosine by BER pathway and glycosylases, i.e., TDG and SMUG1. The other products of 5mC oxidation process, 5fC and 5caC undergo BER as well. 5mC, 5-methylcytosine; 5hmC, 5-hydroxymethylcytosine; 5hmU, 5-hydroxymethyluracil; 5fC, 5-formylcytosine; 5caC, 5-carboxylcytosine; AID, activation-induced deaminase; APOBEC, DNA dC->dU-editing enzyme APOBEC; BER, base excision repair pathway; Cyt, cytosine; DNMT, DNA methyltransferase; SAH, S-adenosyl-L-homocysteine; SAM, S-adenosyl-L-methionine; SMUG1, Single-strand selective monofunctional uracil DNA glycosylase; Thy, thymine; TET, Methylcytosine dioxygenase TET; TDG, G/T mismatch-specific thymine DNA glycosylase.

The mechanism of DNA methylation and demethylation processes have been depicted in Figure 1.

## 4. Curcumin as an Epigenetic Inhibitor of Mammary Cancer

The numerous studies have shown that natural spices and their bioactive components, such as turmeric and curcumin, may induce epigenetic remodeling in breast cancer cells leading to TSG reactivation and oncogene downregulation. Those changes in expression of genes encoding proteins involved in the regulation of intracellular oncogenic signaling pathways may cause inhibition of breast cancer cell proliferation via cell cycle arrest and simultaneously induce cell apoptosis [9,12,15,51].

Several in vitro and in vivo studies on breast cancer indicated that the main bioactive component of turmeric rhizome, curcumin is a potent breast cancer inhibitor possessing anti-proliferative and proapoptotic properties [52,53,54].

The studies in two tumor cell lines, human breast cancer MCF-7 and T cell lymphoma EL4 of murine origin, as well as in two types of normal cells, including mouse spleen lymphocytes and NIH3T3 mouse fibroblast cells, have revealed the potent cytotoxic cancer-specific activity of curcumin. Total curcumin uptake was significantly higher in both tumor cell lines comparing to normal cells. Moreover, localization of curcumin in the human breast cancer MCF-7 cells has been determined using laser confocal microscopy [55]. The different subcellular distribution of curcumin was observed in MCF-7 cells. It has been documented that curcumin accumulates mainly in the cell membrane and the uptake of curcumin by other cell components is in the following order: cytoplasm > nucleus > mitochondria. Most likely, the lipophilic curcumin interacts with cellular membrane lipids, what explains why the polyphenol is mainly located in the cell membrane. These findings are consistent with the results of the other studies showing that curcumin uptake is significantly higher in tumor cells compared to normal cells [55,56,57]. It could be caused by different composition of the lipid droplets in non-malignant and malignant human breast epithelial cell lines. Abramczyk et al. analyzed the chemical composition of lipids and proteins in non-malignant (MCF10A), mildly malignant (MCF7) and malignant (MDA-MB-231) breast cancer cells by Raman imaging. Results of the studies demonstrate increased lipid contents in malignant breast cancer cells compared to non-malignant cells. The number of cytoplasmic lipid droplets correlates with increased aggressiveness of cancer. In malignant breast cells MCF7 and MDA-MB-231 it is respectively 2 and 4 times higher, than in non-malignant MCF10A cells. The higher content of lipid droplets in breast cancer cells may explain better solubility of curcumin and its higher intracellular concentration [58]. Thus, increased uptake of curcumin is consistent with its increased toxicity [55].

### 4.1. Curcumin and DNMTs

Alterations in methylation of gene promoters play an important role in gene transcriptional activity. Hypermethylation-mediated silencing of TSGs and hypomethylation-mediated activation of oncogenes and pro-metastatic genes are probably the most consistent epigenetic hallmarks of human cancers, including breast cancer. Significance of epigenetic changes in cancer development, especially aberrant DNA methylation patterns, is comparable to the relevance of genetic mutations.

The DNA methylation is mediated by specific DNMTs. Increased level of DNMTs was observed in cancer patients. In the study of Mirza et al., the levels of DNMT1, DNMT3A, and DNMT3B mRNA were observed to be 1.2- to 4.4-folds, 1.1- to 3.77-folds, and 1.06- to 4.01-folds elevated in the most of the analyzed breast cancer tissues, respectively, as compared to the adjacent normal breast tissues [59].

In another study, the expression of DNMT1, DNMT3A, and DNMT3B in 256 breast cancer and 36 breast fibroadenoma cases were investigated. The DNMT1 and DNMT3A expression levels were significantly higher in breast cancer than in fibroadenoma samples. The DNMT1 and DNMT3A overexpression was associated with promoter hypermethylation and downregulation of ERα and BRCA1 [60].

The DNMT1 up-regulation was also revealed in most cancer-associated fibroblasts in relation to their corresponding adjacent normal fibroblasts. The ectopic expression of DNMT1 activated primary normal breast fibroblasts and promoted their pro-carcinogenic effects, both in vitro and in orthotopic tumor xenografts whereas DNMT1 knockdown normalized breast myofibroblasts. DNMT1 seems to be critical for the activation of breast stromal fibroblasts as well as the persistence of their active status [61].

Therefore, in recent years, DNA methylation has emerged as an attractive target for the anti-cancer therapeutics. Moreover, natural bioactive compounds, including curcumin, have received increasing attention as potential modulators of epigenetic machinery in cancer cells. The numerous studies have shown that curcumin exerts robust epigenetic anti-cancer effects against breast cancer (Table 2).

In 2009, Liu et al. based on the results of molecular docking (interaction of curcumin and DNMT1) suggested that curcumin covalently blocks the catalytic thiolate of C1226 of DNMT1 to exert its inhibitory effect [62]. Moreover, they observed in in vitro tests that curcumin and one of its major metabolites—tetrahydrocurcumin, can inhibit the activity of CpG Methyltransferase M.SssI (a DNMT1 analog with a structurally similar catalytic domain) [62].

Furthermore, apart from that direct chemical mechanism of the reduction of DNMTs enzymatic activity in response to curcumin exposure, the second one is the biological inhibition of DNMTs synthesis. In many studies, in breast cancer cells incubated with curcumin, the DNMT1 protein level was significantly decreased [59,63,64,65,66] and it was consistent with the mRNA levels of DNMT1, also significantly downregulated in curcumin-exposed cells [59,63,65,66]. These curcumin-mediated effects may be associated with the disruption of binding of the NF-κB/SP1 complex to the *DNMT1* promoter region [66].

### 4.2. Curcumin and HDACs/HATs

Epigenetic alterations, which may occur as a part of the carcinogenesis process, modulate gene expression. In tumor cells, silencing of TSGs and activation of oncogenes are observed and usually, these aberrant methylation patterns are not caused by histone modification or DNA methylation alone. All those epigenetic processes are combined and interdependent [13].

Histone deacetylases (HDACs) and histone acetyltransferases (HATs) are the two major groups of enzymes that modulate chromatin structure via histone modifications. The balance between histone acetylation and deacetylation is important for the epigenetic regulation of gene function and dysregulation of these processes may contribute to cancer development. Curcumin has been reported to inhibit HDACs such as HDAC1 and HDAC2 in breast cancer cell lines, MCF-7 and MDA-MBA-231. The HDAC1 and HDAC2 expression levels were constitutively very high in these breast cancer cell lines, comparing to the normal breast epithelial cells MCF-12F. Treatment of cells with 50 μM curcumin for 24 h led to 84% inhibition of HDAC1 expression and 70% inhibition of HDAC2 expression in the case of MCF-7 cells. Similar exposure to curcumin in MDA-MB-231 cells downregulated HDACs, particularly HDAC2, but to a lesser extent (HDAC1 by 75% and HDAC2 by 45%). These changes were accompanied by cell cycle arrest via P21 upregulation in breast cancer MCF-7 cells, and by apoptosis induction via caspase-9 activation [59,67].

Similar observations were made by Mirza et al., 96 h exposure of MCF-7 and MDA-MB-231 cell to 10 µM curcumin led to a 3-fold decrease in HDAC1 level in both cell lines. A decrease in the HDAC1 (and DNMT1) expression caused an opposite effect on the P21 protein level. Curcumin exposures of the breast cancer cells resulted in 2- and 4-fold increases in the P21 expression in MDA-MB-231 and MCF-7, respectively [59].

Curcumin was also found to be highly potent direct HDAC inhibitor. Molecular docking studies showed that curcumin binds to HDAC8 (the other class I HDAC, apart from HDAC1 and 2) and makes hydrophobic contact with active site residues of the enzyme [72].

### 4.3. Curcumin and miRNAs

Curcumin has also been shown to modulate the expression of miRNAs (small non-coding RNA sequences containing about 22 nucleotides involved in post-transcriptional regulation of gene expression) in breast cancer [73]. Curcumin was able to affect the expression of oncogenic (miR-19a and miR-19b) [68] and tumor-suppressive miRNAs (miR-15a, miR-16, miR-29a, miR-34a, and miR-181b) in breast cancer cells [64,69,70,71]. The observed changes in miRNA expression led in consequence to the suppression of tumorigenesis and metastasis, and induction of apoptosis.

### 4.4. Curcumin Epigenetic Anti-Cancer Effects Revealed in In Vivo Studies

Du et al. revealed that curcumin in MCF-7 cells downregulates the mRNA and protein level of DNMT1, thereby decreasing the methylating activity of the nuclear extract and global DNA methylation in MCF-7 cells. Curcumin reactivates a silenced TSG ras-association domain family protein 1A (RASSF1A) at least partially due to its promoter hypomethylation in breast cancer MCF-7 and MDA-MB-231 cell lines. Since *RASSF1A* tumor suppressor silencing is associated with the deregulated proliferation activity of some cancer cells, the curcumin-mediated RASSF1A reactivation may be associated with its anti-proliferative activity in vitro and anti-tumor growth activity in vivo. The mRNA level of *RASSF1A* was found to be significantly higher not only in breast cancer cell lines but also in MCF-7 cell engrafted tumor tissue collected from tumor bearing nude mice treated with an intraperitoneal administration of 100 mg/kg curcumin in reference to those treated with the vehicle. Curcumin treatment caused also 65% decrease in tumor size in nude mice without any observed cytotoxicity [66].

The antitumor activity of curcumin in ER-negative human breast cancer was assessed in in vivo mouse model of breast cancer (MDA-MB-231 xenograft model in female Foxn1nu/nu mice). 16 mice were randomized into two groups: 8 controls (normal diet) and 8 curcumin-treated through 6 weeks after tumor cell implementation. Results obtained in the study indicated that curcumin inhibits tumor growth and angiogenesis without toxicity effect on mice. The data showed that curcumin represses the activation of NF-κB as far as NF-κB-regulated gene products such as cyclin D1, p65, and PECAM-1 (significant reductions in the expression of PECAM-1, cyclin D1, and p65 compared to the control group were observed) [74]. Similar observations were made in the other study, in which the effects of curcumin on the human breast cancer cell line MDA-MB-231 in vitro and in a mouse metastasis model (MDA-MB-231 cells were intracardiac injected in immunodeficient mice) were examined. Curcumin appeared to inhibit the expression and activity of AP-1 and NF-κB and consequently reduce the expression of major matrix metalloproteinases (MMPs). Curcumin caused also a diminution of ΙκΒ and p65 phosphorylation, reduced activation of survival pathway NF-κB and the number of metastases [75].

Curcumin alone and in combination with mitomycin C (MMC) inhibited MCF-7 breast cancer cell proliferation and viability in vitro and in vivo. In MCF-7 xenografts, combined administration of curcumin (100 mg/kg) and MMC (1–2 mg/kg) for 4 weeks produced significantly greater inhibition on tumor growth than either treatment alone. The combined treatment resulted in significantly greater G1 arrest than MMC or curcumin alone. Moreover, the cell cycle arrest was associated with inhibition of cyclin D1, cyclin E, cyclin A, cyclin-dependent kinase 2 (CDK2) and CDK4, along with the induction of the cell cycle inhibitor P21 and P27 both in MCF-7 cells and in MCF-7 xenografts. These proteins were regulated through the P38 MAPK pathway [76].

### 4.5. Curcumin Epigenetic Anti-Cancer Effects Revealed in Clinical Trials

Since there is a need to improve the efficacy of breast cancer chemotherapy especially with safe molecules, therefore the feasibility and tolerability of the combination of chemotherapeutics with natural compounds are investigated.

In a clinical trial evaluating curcumin in combination with docetaxel in advanced and metastatic breast cancer patients, it was revealed that the safety profile of the combination is consistent with that observed with monotherapy of docetaxel. Curcumin was given orally for seven consecutive days in 8000 mg/day dose, in combination with docetaxel 100 mg/m^2^ administered every 3 weeks for six cycles. Curcumin/docetaxel combination demonstrated antitumor activity. Among the 14 patients enrolled in this study, nine patients were evaluated for tumor response. In seven patients, the biological response was documented with the decrease of tumor markers, which was up to 50% in four patients [77].

In the other clinical trial the efficacy and safety of curcumin, administering intravenously, in combination with paclitaxel in patients with advanced, metastatic breast cancer was explored. A total of 150 women with advanced and metastatic breast cancer were randomly assigned to receive either paclitaxel (80 mg/m^2^) plus placebo or paclitaxel plus curcumin (CUC-1^®^, 300 mg solution, once per week) intravenously for 12 weeks with 3 months of follow-up (133 patients complete the study treatments). Objective response rate (percent of patients with complete and partial tumor reduction) of curcumin/paclitaxel combination was significantly higher (50.7%) than that of the placebo/paclitaxel (33.3%; *p* < 0.05) after 12 weeks of treatment and 4 weeks of follow-up. Intravenously administered curcumin caused no major safety issues and no reduction in quality of life, and it was assumed as slight beneficial in reducing fatigue [78].

Those findings confirmed the safety of curcumin and its potential in elevating antitumor activity of conventional chemotherapeutics applied in anti-cancer treatment, also in breast cancer therapy.

## 5. Insight into the Other Bioactive Components of Turmeric Rhizome as Potential Epigenetic Modifiers

**Figure 2:**
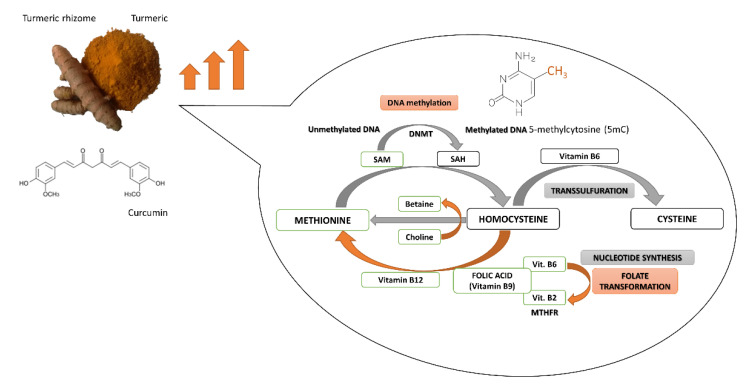
Potential interference of curcumin and other components of turmeric in DNA methylation reaction and one-carbon metabolism. MTHFR, Methylenetetrahydrofolate reductase; SAH, S-adenosyl-L-homocysteine; SAM, S-adenosyl-L-methionine.

The turmeric rhizome contains the numerous bioactive agents, including not only polyphenolic curcumin but also the B-group vitamins (riboflavin, pyridoxine and folic acid), antioxidative vitamins C and E, several minerals (i.e., zinc), and turmeric oil [19]. Folic acid as a dietary methyl donor, as well as vitamins B2 and B6 as cofactors of enzymes, are implicated in one-carbon metabolism involved in SAM synthesis and DNA methylation reaction. The bioactive compounds derived from turmeric rhizome may affect DNA methylation processes by changes in DNMTs expression and activity, as well as by alterations in SAM level, the ubiquitous donor of a methyl group in DNA methylation reaction (Figure 2).

DNA methylation is a SAM-dependent modulation in which SAM plays the role of methyl group donor. The process of SAM synthesis (from methionine) is a part of the methionine cycle [79]. In the cycle, SAM is converted to S-adenosylhomocysteine (SAH) that is transformed to homocysteine. Subsequently obtaining methyl group from 5-methyltetrahydrofolate (5-MTHF) enables the methylation of homocysteine to methionine and then the formation of SAM. Importantly, 5-methyltetrahydrofolate is created in the folate cycle that begins with the conversion of folic acid. The connection of folate and methionine cycles allows transferring a methyl group from 5-MTHF to methionine [80].

After carrying out the genome methylation, SAM is converted to SAH that is a potent inhibitor of DNMTs, especially DNMT1. SAH can bind to the catalytic region of most SAM-dependent methyltransferases [79]. The ratio of SAM to SAH is called “methylation index” [81]. The proper ratio of SAM to SAH is very important for the stability of DNA methylation patterns. Methylation index can indicate the probability of DNA hyper or hypomethylation [82]. Cancer development is associated with SAM depletion, global DNA hypomethylation, activation of oncogenes as well as hypermethylation and silencing of TSGs [83,84,85].

As mentioned above, the methionine cycle is connected with a folate cycle. The folate cycle is regulated by B-group vitamins which, as cofactors of the cycle, indirectly affect the synthesis and concentration of SAM. One carbon metabolism pathway starts with folic acid (vitamin B9) which is transformed into dihydrofolate (DHF), tetrahydrofolate (THF), 5,10-methylenetetrahydrofolate (5,10-MTHF) and 5-MTHF [86]. Conversion of THF to 5-MTHF occurs in the presence of pyridoxine (vitamin B6), while riboflavin (vitamin B2) as a component of FAD, participates in the conversion of 5,10-MTHF to 5-MTHF. A low pyridoxine plasma concentration is linked to an increase in SAH level, a lower ratio of SAM to SAH and DNA hypomethylation, which can increase the risk of cancer. According to the literature, vitamins B9 and B6 take part in the reduction of breast cancer growth [13]. They regulate indirectly the level of SAM and SAH, while SAH is an inhibitor of DNMTs.

Moreover, folic acid can enhance curcumin inhibitory effect on DNMTs activity, probably by improving the solubility and bioavailability of curcumin, what was documented by Liu Z [87]. The important role of folates is confirmed by studies including Chinese breast cancer cases. The studies indicate that high folate intake may reduce cancer risk in premenopausal women. The protective role of folate in breast cancer, particularly in ER-negative cancer was shown in another prospective study [88]. On the other hand, some cases indicated that low methionine and B-vitamins intake may be related to a breast cancer carcinogenesis [89]. Other studies showed that folate deficiency leads to decreased SAM level and slight, statistically significant global hypomethylation which is an early epigenetic modification in many cancers [90].

As it was mentioned before, antioxidant and anti-inflammatory properties are also the two mechanisms of curcumin chemopreventive activity. Accumulation of free-radicals (reactive oxygen species (ROS) and reactive nitrogen species (RNS)) is responsible for peroxidation of membrane lipids and oxidative damage of DNA and proteins, and it is implicated in the development of chronic pathological complications, such as cancer. Curcumin has been shown to inhibit cyclooxygenase-2 (COX-2), lipoxygenase (LOX), inducible nitric oxide synthase (iNOS) and xanthine oxidoreductase (XOR) enzymes, generating ROS. That is the reason why curcumin is considered as a potent chemopreventive antioxidant agent [91].

Additionally, curcumin enhances cell antioxidant ability via increasing the activity of antioxidant enzymes such as glutathione peroxidase (GPx), glutathione reductase (GR), glutathione S-transferase (GST) and superoxide dismutase (SOD), glucose-6-phosphate dehydrogenase and catalase [92,93].

It was also reported that curcumin mediates nuclear transport of transcription factor NF2L2 (Nrf-2; Nuclear factor erythroid 2-related factor 2) which regulates antioxidant signaling pathway. NF2L2-targeted genes encode proteins that may be classified as the phase II xenobiotic-metabolizing antioxidant enzymes, playing a pivotal role in cancer prevention [94].

However, curcumin can also activate ROS formation and enhance oxidative stress in cancer cells. Curcumin-mediated rapid generation of ROS induces apoptosis via caspase activation and alterations in mitochondrial membrane potential followed by cytochrome c release. Thus, curcumin can initiate apoptosis and mediate chemosensitization of cancer cells [57,95].

In human breast cancer cells, a combination of arabinogalactan and curcumin significantly decreased cell growth without any significant effect on normal cells. This combination of compounds promoted apoptosis by increased ROS level, changed mitochondrial membrane potential and glutathione reduction. Moreover, in vivo mice studies indicated that the combination of curcumin and arabinogalactan inhibited the progression of breast tumors [96].

Interestingly, the turmeric rhizome also contains two potent antioxidants: vitamin C and vitamin E. The vitamins may intensify the antioxidative properties of curcumin by neutralizing free radicals of environmental carcinogens. Moreover, according to Minor’s studies, vitamin C participates in the hydroxylation of 5-methylcytosine to 5-hydroxymethylcytosine in DNA and it probably takes part in the modulation of epigenetic control of genome activity leading to the active demethylation of DNA [97].

It is also necessary to mention about two important substances that are present in a turmeric rhizome: zinc ions and turmeric oil. Some studies show that zinc deficiency may result in reduced methyl group transfer from SAM to cytosine in the methylated gene. It can also complicate the binding of DNMT1 to DNA, which can lead to DNA demethylation and global DNA hypomethylation [98].

## 6. Discussion, Conclusions and Future Perspectives

**Figure 3:**
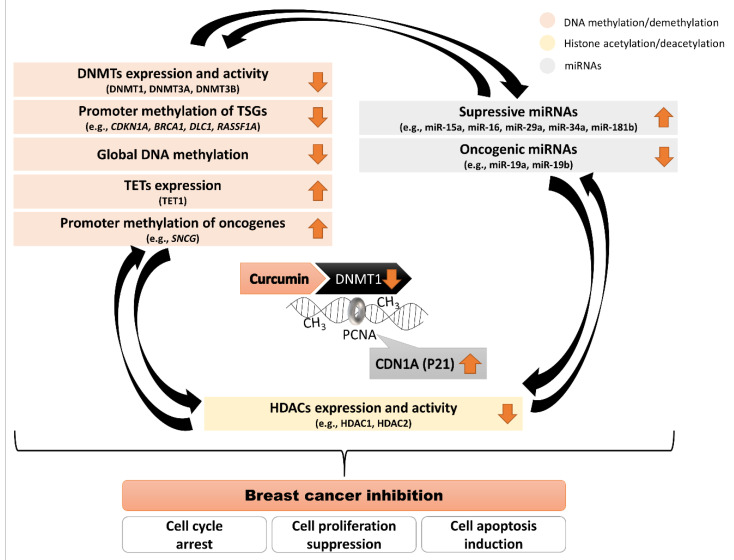
Scheme demonstrating mechanisms used by curcumin to drive changes in the epigenome in breast cancer inhibition. Curcumin binds to the DNMT1 catalytic domain and impairs its enzymatic activity. PCNA is crucial for DNMT1 activity during replication when DNA methylation pattern is copied from a parental to a daughter DNA strand. CDN1A (P21 encoded by *CDKN1A* gene) competes with DNMT1 for the same binding site on PCNA, which impairs DNMT1 activity. Curcumin that leads to an increase in P21 expression may affect DNA methylation. Interconnections between the components of the epigenome: DNA methylation, histone modifications and miRNAs. Curcumin driving changes in DNA methylation patterns in breast cancer cells may have indirect effects on other epigenetic components (histone modifications and miRNAs) and vice versa.

Numerous data have shown that curcumin from turmeric rhizome can modulate our epigenome (Figure 3). It raises questions on its pharmacological applications in epigenetic chemoprevention and anti-cancer therapy. From the experimental evidence discussed in the present review, epigenetic modifications, mainly DNA methylation, are one of the mechanisms by which curcumin inhibits breast cancer cell growth.

Aberrant epigenetic processes lead to dysregulated expression of many genes, with the upregulated oncogenes and silenced TSGs at the different stages of breast cancer development. Curcumin binds to the DNMT1 catalytic domain and impairs its enzymatic activity. Moreover, curcumin-mediated CDN1A (P21 protein encoded by *CDKN1A* gene) upregulation impairs DNMT1 activity. Since PCNA is crucial for DNMT1 activity during replication when DNA methylation pattern is copied from a parental to a daughter DNA strand, curcumin-reactivated CDN1A (P21) competes with DNMT1 for the same binding site on PCNA. Hence, curcumin that leads to a relevant increase in P21 expression may affect DNA methylation processes. The hitherto studies in different breast cancer models have shown that curcumin exposure caused relevant changes in the expression profile of genes encoding DNA methylating/demethylating enzymes (DNMTs (mostly DNMT1) downregulation and TET1 upregulation), and histone deacetylating enzymes (HDAC1 and HDAC2 downregulation), and alterations in the activity of numerous miRNAs. Therefore, the interconnections between the components of the epigenome: DNA methylation, histone modifications and miRNAs have to be highlighted. Curcumin driving changes in DNA methylation patterns in breast cancer cells may have indirect effects on other epigenetic components (histone modifications and miRNAs) and vice versa.

Recent studies have shown that, as compared to most single-target drugs, the majority of bioactive compounds, including curcumin do not target a single protein or pathway but affects multiple cell signaling pathways. It may allow phytochemicals such as curcumin to evade the development of resistance due to the activation of supporting alternative pathways. Curcumin via epigenetic anti-cancer activity may lead to re-activation of numerous DNA-methylation-silenced TSGs and downregulation of oncogenes via promoter hypermethylation, and the proteins encoded by these genes might be the negative or positive regulators of various intracellular oncogenic signaling pathways. Moreover, curcumin by modulating the activity of different transcription factors, such as AP-1, NF-κB/SP1 or TP53, may affect the transcriptional activity of *DNMT1*, as the AP-1, NF-κB/SP1 or TP53 binding sites have been identified within the DNMT1 regulatory region. It seems like the curcumin would have the potential to alter “cancer-specific” DNA methylation pattern and gene expression profile towards “normal-like”. Cell-type-specific effects of curcumin are remarkable in many types of cancer, including breast cancer and only continued research can allow a better understanding of cell signaling pathways targeted by this potent anti-cancer agent.

Thus, curcumin showed promising chemopreventive effects in the laboratory experiments, but its clinical application is still limited because of low water solubility and low metabolic stability. Nevertheless, the numerous studies undergoing in various laboratories and clinics intensively working on evolving novel phytochemical-based treatment options for breast cancer implies that phytochemicals such as curcumin are the leading molecules for future anti-cancer drug development targeting breast cancer-related signaling pathways. Moreover, the effective doses of curcumin have not been shown to exert any toxicities or side effects making this bioactive compound ideal preventative and anti-cancer agent.

Thus, the main challenges in the investigation of curcumin as an epigenetic anti-cancer agent are new strategies of increasing its bioavailability, assessing the efficacy of the metabolites and determining the role of this phytochemical alone or in combination with other turmeric-derived compounds and existing drugs in improving anti-cancer efficacy. These important issues should be addressed in future nutriepigenomic studies. Whilst there is much more to be done, the available data so far indicate that curcumin can be a potential target of drug development against cancer.

## Figures and Tables

**Table 1:** Nutrients in 100 g of turmeric, *Curcuma longa* L. (FoodData Central: Spices, turmeric, ground; Data Type: SR Legacy; Food Category: Spices and Herbs; FDC ID: 172231; NDB Number: 2043; FDC Published: 4/1/2019; U.S. Department of Agriculture (USDA), Agricultural Research Service [19].)

Name	Amount (Min-Max)	Unit
Water	12.850	g
Energy	312.000	kcal
Protein	9.680	g
Total lipid (fat)	3.250	g
Carbohydrate	67.140	g
Fiber, total dietary	22.700	g
Calcium, Ca	168.000	mg
Iron, Fe	55.000	mg
Magnesium, Mg	208.000	mg
Phosphorus, P	299.000	mg
Potassium, K	2080.000	mg
Sodium, Na	27.000	mg
Zinc, Zn	4.500	mg
Copper, Cu	1.300	mg
Manganese, Mn	19.800	mg
Selenium, Se	6.200	µg
Vitamin C, total ascorbic acid	0.700	mg
Vitamin B_1_ (thiamin)	0.058	mg
Vitamin B_2_ (riboflavin)	0.150	mg
Vitamin B_3_ (niacin)	1.350	mg
Vitamin B_5_ (pantothenic acid)	0.542	mg
Vitamin B_6_ (pyridoxine)	0.107 (0.034–0.180)	mg
Folate, total	20.000	µg
Choline, total	49.200	mg
Betaine, total	9.700	mg
Vitamin E (alpha-tocopherol)	4.430	mg
Vitamin K (phylloquinone)	13.400	µg
Fatty acids, total saturated	1.838	g
Fatty acids, total monounsaturated	0.449	g
Fatty acids, total polyunsaturated	0.756	g
Curcuminoids	2.000–9.000	g

**Table 2:** Curcumin impact on epigenetic machinery in breast cancer inhibition.

DNMTs Expression	Change	Model	Curcumin Treatment	Reference
mRNA level				
	decrease in all DNMTs (DNMT1, DNMT3A, DNMT3B)	MCF-7 MDA-MB-231	IC50—10 µM/96 h	Mirza S. et al., J Breast Cancer, 2013 [59]
	decrease in all DNMTs (DNMT1, DNMT3A, DNMT3B)	MCF-7	2 and 20 µM/12 and 24 h	Chatterjee B. et al., J Cell Biochem, 2019 [63]
	decrease in DNMT1 (without changes in DNMT3A, DNMT3B)	MDA-MB-361 MDA-MB-231 MCF-7	40 µM/48 h	Liu Y. et al., Mol Cell Biochem, 2017 [65]
	decrease in DNMT1	MCF-7	10 and 20 µM/72 h	Du L. et al., Nutr Cancer, 2012 [66]
protein level				
	2-fold decrease in DNMT1	MCF-7 MDA-MB-231	IC50—10 µM/96 h	Mirza S. et al., J Breast Cancer, 2013 [59]
	decrease in all DNMTs (DNMT1, DNMT3A, DNMT3B)	MCF-7	2 and 20 µM/12 and 24 h	Chatterjee B. et al., J Cell Biochem, 2019 [63]
	reduction in DNMT1 protein level increase in DNMT3A and DNMT3B protein level	HCC-38 UACC-3199 T47D	5 and 10 µM/6 days	Al-Yousef N. et al., Oncol Rep, 2020 [64]
	decrease in DNMT1 (without changes in DNMT3A, DNMT3B)	MDA-MB-361 MDA-MB-231	40 µM/48 h	Liu Y. et al., Mol Cell Biochem, 2017 [65]
	decrease in DNMT1	MCF-7	10 and 20 µM/72 h	Du L. et al., Nutr Cancer, 2012 [66]
Other proteins				
	increase in *TET1* mRNA and TET1 protein level	HCC-38	5 and 10 µM/6 days	Al-Yousef N. et al., Oncol Rep, 2020 [64]
	3-fold decrease in HDAC1 protein level	MCF-7 MDA-MB-231	IC50—10 µM/96 h	Mirza S. et al., J Breast Cancer, 2013 [59]
	decrease in HDAC1 and HDAC2 protein level	MCF-7 MDA-MB-231	50 µM/24 h	Mukherjeea S. et al., Int. J. Green Nanotechnol, 2012 [67]
oncogene	decrease in *SNCG* mRNA (down to 2-fold) and SNCG protein level	T47D HCC-38	5 and 10 µM/6 days	Al-Yousef N. et al., Oncol Rep, 2020 [64]
tumor suppressor	induction of *DLC1* expression on mRNA and protein level	MDA-MB-361	20 and 40 µM/48 h	Liu Y. et al., Mol Cell Biochem, 2017 [65]
tumor suppressor	increase in *BRCA1* mRNA level up to 2-fold with consequent high increase in BRCA1 protein level	HCC-38 UACC-3199	5 and 10 µM/6 days	Al-Yousef N. et al., Oncol Rep, 2020 [64]
tumor suppressor	increased level of *CDKN1A* (*p21*, 2-fold in MDA-MB-231 and 4-fold in MCF-7)	MCF-7 MDA-MB-231	IC50—10 µM/96 h	Mirza S. et al., J Breast Cancer, 2013 [59]
tumor suppressor	increased level of *CDKN1A (p21)*	MCF-7 MDA-MB-231	50 µM/24 h	Mukherjeea S. et al., Int. J. Green Nanotechnol, 2012 [67]
tumor suppressor	increased expression of *TP53* and *KLF4* on mRNA and protein levels	MCF-7	2 and 20 µM/12 and 24 h	Chatterjee B. et al., J Cell Biochem, 2019 [63]
tumor suppressor	enhanced mRNA and the protein levels of *RASSF1A*	MCF-7 MDA-MB-231	10 and 20 µM/72 h	Du L. et al., Nutr Cancer, 2012 [66]
transcription factor	reduction in *SP1* expression	MDA-MB-361	40 µM/48 h	Liu Y. et al., Mol Cell Biochem, 2017 [65]
DNMTs activity				
	methylation activity of DNMT1 in nuclear extract decreased by about 70% (compared to the control)	MCF-7	10 and 20 µM/72 h	Du L. et al., Nutr Cancer, 2012 [66]
Promoter methylation				
	demethylation of the proximal promoter of *CDKN1A* (*p21*)	MCF-7	2 and 20 µM/12 and 24 h	Chatterjee B. et al., J Cell Biochem, 2019 [63]
	hypermethylation of the *SNCG* promoter	T47D	5 and 10 µM/6 days	Al-Yousef N. et al., Oncol Rep, 2020 [64]
	partial hypomethylation of the *BRCA1* promoter	HCC-38 UACC-3199	5 and 10 µM/6 days	Al-Yousef N. et al., Oncol Rep, 2020 [64]
	demethylation of *DLC1* promoter	MDA-MB-361	20 and 40 µM/48 h	Liu Y. et al., Mol Cell Biochem, 2017 [65]
	decrease in *RASSF1A* promoter methylation	MCF-7	10 µM/72 h	Du L. et al., Nutr Cancer, 2012 [66]
Global DNA methylation				
	hypomethylation	MCF-7	2 and 20 µM/12 and 24 h	Chatterjee B. et al., J Cell Biochem, 2019 [63]
	the global DNA methylation (GDM) decreased by about 30–35%	MCF-7	10 µM/72 h	Du L. et al., Nutr Cancer, 2012 [66]
miRNA				
	downregulation of oncogenic miR-19 (modulates downstream proteins: PTEN, AKT1, MDM2, TP53)	MCF-7	1 µM/4 days	Li X. et al., Phytother Res, 2014 [68]
	upregulation of miR-29b	T47D	5 and 10 µM/6 days	Al-Yousef N. et al., Oncol Rep, 2020 [64]
	upregulation of miR-34a (reduction in *BCL2* and *BMI1* expression)	MDA-MB-231 MDA-MB-435	30 or 34 μM/24 h	Guo J. et al., Mol Cell Biochem, 2013 [69]
	upregulation of miR181b (reduction in *CXCL1*, *CXCL2*, *MMPs* expression)	MDA-MB-231	25 μM/24 h	Kronski E. et al., Mol Oncol, 2014 [70]
	upregulation of miR-15a and miR-16 (reduction in *BCL2* expression)	MCF-7	10–60 μM/24 h	Yang J. et al., Med Oncol, 2010 [71]

AKT1 (AKT Serine/Threonine Kinase 1); BCL2 (BCL2 Apoptosis Regulator); BMI1 (BMI1 Proto-Oncogene, Polycomb Ring Finger); BRCA1 (BRCA1 DNA Repair Associated); CDKN1A (Cyclin Dependent Kinase Inhibitor 1A); CXCL1 (C-X-C Motif Chemokine Ligand 1); CXCL2 (C-X-C Motif Chemokine Ligand 2); DLC1 (DLC1 Rho GTPase Activating Protein); DNMT1 (DNA Methyltransferase 1); DNMT3A (DNA Methyltransferase 3 Alpha); DNMT3B (DNA Methyltransferase 3 Beta); HDAC (Histone Deacetylase); KLF4 (Kruppel Like Factor 4); MDM2 (MDM2 Proto-Oncogene); MMPs (Matrix Metallopeptidases); PTEN (Phosphatase And Tensin Homolog PTEN); RASSF1 (Ras Association Domain Family Member 1; Tumor Suppressor Protein RDA32); SNCG (Synuclein, Gamma (Breast Cancer-Specific Protein 1)); SP1 (Sp1 Transcription Factor); TET1 (Tet Methylcytosine Dioxygenase 1); TP53 (Tumor Protein P53).
